# Indoor nocturnal noise is associated with body mass index and blood pressure: a cross-sectional study

**DOI:** 10.1186/s12889-021-10845-2

**Published:** 2021-04-28

**Authors:** Sha Li, Daniel Yee Tak Fong, Janet Yuen Ha Wong, Bradley McPherson, Esther Yuet Ying Lau, Lixi Huang, I. P. Mary Sau Man

**Affiliations:** 1 School of Nursing, Li Ka Shing Faculty of Medicine, The University of Hong Kong, 21 Sassoon Road, Pokfulam, Hong Kong, China; 2 Division of Speech and Hearing Sciences, Faculty of Education, The University of Hong Kong, Pokfulam, Hong Kong, China; 3 Sleep Laboratory, Department of Psychology, The Education University of Hong Kong, 10 Lo Ping Road, Tai Po, New Territories Hong Kong, China; 4 Centre for Psychosocial Health, The Education University of Hong Kong, 10 Lo Ping Road, Tai Po, New Territories Hong Kong, China; 5 Department of Mechanical Engineering, The University of Hong Kong, Pokfulam, Hong Kong, China; 6 Department of Medicine, Li Ka Shing Faculty of Medicine, The University of Hong Kong, 21 Sassoon Road, Pokfulam, Hong Kong, China

**Keywords:** Indoor noise, Obesity, Body mass index, Blood pressure

## Abstract

**Background:**

Studies have demonstrated that noise is associated with various health problems, such as obesity and hypertension. Although the evidence of the associations of noise with obesity and hypertension is inconsistent, there seems to be a stronger association of the latter. This study aimed to investigate the associations of noise with body mass index (BMI) and blood pressure in adults living in multi-story residential buildings.

**Methods:**

A cross-sectional study was conducted in Hong Kong from February 2018 to September 2019. The Weinstein Noise Sensitivity Scale, Pittsburgh Sleep Quality Index, ENRICHD Social Support Instrument, Patient Health Questionnaire, Perceived Stress Scale, and Hospital Anxiety and Depression Scale were administered to the participants. BMI and blood pressure were assessed. Nocturnal noise exposure and total sleep duration were measured for a week.

**Results:**

Five hundred adults (66.4% female), with an average age of 39 years (range: 18–80), completed the study. The average levels of nocturnal noise, BMI, systolic blood pressure (SBP), and diastolic blood pressure (DBP) were 51.3 dBA, 22.2 kg/m^2^, 116.0 mmHg, and 75.4 mmHg, respectively. After adjusting for sociodemographic characteristics, nocturnal noise was associated with BMI (b = 0.54, 95% CI: 0.01 to 1.06, *p* = 0.045) and SBP (b = 2.90, 95% CI: 1.12 to 4.68, *p* = 0.001). No association was detected between nocturnal noise and DBP (b = 0.79, 95% CI: − 0.56 to 2.13, *p* = 0.253). Specifically, higher nocturnal noise was associated with higher BMI (b = 0.72, 95% CI: 0.07 to 1.38, *p* = 0.031) and SBP (b = 3.91, 95% CI: 2.51 to 5.31, *p* < 0.001) in females but only higher SBP (b = 3.13, 95% CI: 1.35 to 4.92, p < 0.001) in males. The association between noise and SBP remained significant (b = 2.41, 95% CI: 0.62 to 4.20, *p* = 0.008) after additionally adjusting for lifestyle, diagnosis of hypertension, psychometric constructs, and sleep.

**Conclusions:**

Indoor nocturnal noise was associated with BMI and blood pressure in females but only blood pressure in males. It is important to control nocturnal noise or use soundproofing materials in buildings to reduce noise exposure.

## Background

In recent decades, environmental noise pollution has gained attention owing to its adverse impacts, such as cardiovascular and metabolic effects on human health [1]. Among noise at different time periods during a day, nocturnal noise has been noted to have the strongest association with health problems [2]. Cardiovascular and metabolic outcomes of noise, e.g., hypertension, ischemic heart diseases, stroke, diabetes, and obesity, have been reported to be associated with noise exposure in some studies, as summarized by Kempen et al. [1]. For instance, an effect of noise (L_night_, outside) on hypertension was proposed to start to occur when the noise level was above 50 dBA [3]. Despite that there was no sufficient evidence of a threshold for the effect of noise on obesity, there might be a threshold around 45–50 dB (L_den_) in the association between noise and waist circumference (WC) [4].

Hypertension and obesity are risk factors for cardiovascular disease (CVD), which is the leading cause of death globally [5–7]. At least 2.8 million and 17.9 million people die each year due to overweight or obesity and from CVD, respectively [5, 8]. There is a clear link between obesity and hypertension [9–11]. Specifically, increases in weight, body mass index (BMI), WC, waist-to-hip ratio (WHR), and waist-to-height ratio were associated with a higher incidence of hypertension [9, 10].

Some other variables have also been identified to play important roles in the association between noise and non-auditory effects. One of them is noise sensitivity. It is suggested that people who are less tolerant of noise are more vulnerable to the negative effects of noise and experience more noise-related health problems [12, 13], and it was noise sensitivity that predicts the non-auditory effects of noise instead of noise level [14]. Moreover, the difference in the impact of noise on human beings has also been detected in specific subgroups, such as in different genders [15]. Additionally, the interaction of noise with lifestyle, such as consumption of alcohol, has also been reported [16].

Furthermore, a major effect of noise on physical health is assumed to be related to sleep [2], which is important in regulating hormonal, glucose, and cardiovascular function [17]. Long-time exposure to noise can cause annoyance and sleep disturbances [2], with these two factors associated with increased activity in the hypothalamic-pituitary-adrenal (HPA) axis [2]. This increased activity leads to the elevation of stress hormones such as cortisol and hence, an imbalance in stress regulation [18, 19]. Elevated cortisol levels are associated with fat accumulation in visceral depots [20], impaired glucose regulation [21], and impact BMI, WC, and blood pressure [22]. A previous study showed that general adiposity, measured by BMI, was associated with an increased death rate [23]. Moreover, there is increasing evidence that both quantitative and qualitative sleep disturbances could result in obesity and hypertension [24–26].

The evidence on the effect of noise on blood pressure is inconsistent, and relatively fewer studies analyzed the role of noise on obesity or BMI when compared with hypertension [1]. The WHO Environmental Noise Guidelines have rated the evidence on the association of noise and hypertension as very low due to a response rate of less than 60% reported in many of the studies and the self-reported nature of hypertension measure [1]. Despite that recent studies incorporating cohort studies with moderate quality showed that exposure to noise increased the risk of hypertension, most of the studies were conducted in Europe [27, 28]; focused on aircraft, transportation, or occupational noise; and targeted participants residing in detached, semi-detached, or terraced houses [29]. Moreover, most of the studies have not measured but have estimated or modeled noise levels, which are affected by the bedroom façade, opening of windows, and sound insulation of the house [2]. However, the indoor noise level is determined by multiple factors and not just by outdoor noise, such as traffic noise [30]. In high-density and modern cities such as Hong Kong, most people live in multi-story residential buildings. It was reported that people living in tall buildings experience reverberation noise from activities in adjacent flats in addition to traffic noise, which might be different from European neighborhood environment modeled noise [31]. Moreover, they may also be exposed to sound sources such as public address system and domestic equipment [31]. Recently, a study focused on multi-story residential buildings in Korea, but the noise levels were still estimated rather than measured [29]. Lastly, potential moderators and mediators such as noise sensitivity and sleep were not well studied in a community setting.

Therefore, this study aimed to investigate the effect of nocturnal noise on BMI and blood pressure in Hong Kong Chinese adults residing in urbanized areas. The primary hypothesis was that nocturnal noise was positively associated with BMI and blood pressure. The secondary hypothesis was that noise sensitivity and some lifestyle factors would moderate the associations between noise and BMI as well as blood pressure, while objective sleep parameters and subjective sleep quality would mediate the associations. Moreover, the moderating effects of sociodemographic and lifestyle characteristics were also assessed.

## Methods

### Design

We carried out this cross-sectional household survey from February 2018 to September 2019 in Hong Kong. First, we obtained a sample list based on the recording from the Hong Kong Census and Statistics Department. All the household address records were organized in quarters and stratified by types of quarters and geographical districts [32]. A random sample of quarters was obtained by applying fixed sampling intervals and non-repetitive random numbers [32]. Every household residing in the selected quarters was covered in the household survey. We mailed the notification letters, which listed the study details, planned visit times, and interviewer identities, to the targeted households before household visits [32]. The interviewer assessed the eligibility criteria during the first household visit. After written informed consent was signed, physical measurements were taken by the interviewer, and the participants were asked to fill in a battery of questionnaires by themselves and to take the nocturnal noise level and objective sleep duration recording for a week. Participants who had completed the survey questionnaires and all the measurements received shopping coupons of HK$300 (approximately US$40) [32].

This study was approved by the Institutional Review Board of the University of Hong Kong/Hospital Authority Hong Kong West Cluster (Reference Number: UW 17–011).

### Participants

Adults (aged 18 years and above) who could read and understand Chinese were eligible for recruitment. As only one participant could be recruited per household, in households with two or more eligible subjects, the one with an upcoming birthday was selected. Adults who were deaf, needed hearing aids, took sleeping pills or other medications for sleep disorders or had a psychiatric illness were excluded [32]. Moreover, people were not recruited if they were pregnant, or had children under 2 years of age, or could not complete the nocturnal noise level and objective sleep duration recording [32]. The sample size required, which was calculated based on a rule of thumb of 10 participants per variable, was 210 participants. Furthermore, increases of 0.39 kg/m^2^ and 0.30 mmHg induced by per 10 dBA increase of noise level were taken for the associations between noise and BMI and systolic blood pressure (SBP), respectively, based on the previous studies [33, 34], with 5% type I error along with a power of 0.8 [35], 351 and 439 participants would be adequate. Lastly, a conservative error of 0.1 on the interaction effect between noise and noise sensitivity was assumed, which yielded a minimum sample size of 396. Therefore, the targeted sample size was 500.

### Measurements

#### General information

We assessed sociodemographic characteristics, including age, sex (Male; Female), marital status (Single; Married/Cohabiting; Separated/Divorced/Widowed), education (Primary school or below; Secondary; Bachelor or above), occupation (Working; Not working; Retired; Students), and family income (from < HK$5000 to > HK$50000) [32]. House type (Multi-story buildings; Townhouse), diagnosis of hypertension (Yes; No), medications taking for hypertension (Yes; No); exercise (from no to 5 h or more/week), smoking (Never; Quit; Yes), and alcohol consumption (Never; Quit; Yes) were also investigated.

#### Physical measurements

Blood pressure, including SBP and diastolic blood pressure (DBP), was measured using an automatic blood pressure monitor (Model HEM-7111, Omron Healthcare Co., Japan). Blood pressure measurements were taken during the household visit, with the participant in a sitting position and with the right arm at the heart level. The interviewer performed two measurements on each participant at a five-minute interval. A third measurement was conducted after 30 min if any recorded SBP was greater than 140 mmHg or the DBP was greater than 90 mmHg. The final SBP and DBP were calculated by taking the average values of the measurements.

Body height and weight were reported by the participants. BMI was calculated as the individual weight in kilograms divided by the square of height in meters.

#### Nocturnal noise measurements

The noise dosimeter (Spark 706RC, Larson Davis Inc., US) or the sound level meter (NSRT Mk2, Convergence Instruments, Canada), which was calibrated with CAL150 (Larson Davis Inc., US) at 94 dB sound pressure level, was placed beside the bed in each participant’s bedroom to record the daily nocturnal noise levels from 12 am to 8 am for a week. A-weighted energy equivalence level with 1-min intervals was recorded.

#### Weinstein noise sensitivity scale (WNSS)

The WNSS, which emphasizes individuals’ affective reactions to noise, was originally designed to measure noise sensitivity [36]. The traditional Chinese WNSS, with 18 items, is reliable and valid to estimate noise sensitivity [37]. All the items were rated from 1 to 6, yielding a total score ranging from 18 to 108. We then standardized the score on a 0–100 scale. Higher total scores indicate greater sensitivity to noise. Cronbach’s alpha of the traditional Chinese WNSS was 0.83 [37].

#### Total sleep duration

Objective total sleep duration was measured using ActiGraph Link and calculated using Cole-Kripke algorithm with the ActiLife (ActiGraph, US). Total sleep time was the total duration scored as “asleep” [38]. The ActiGraph Link was worn on the non-dominant wrist for seven consecutive days, and records of at least four weekdays and 1 weekend day were considered valid.

#### Pittsburgh sleep quality index (PSQI)

The 19-item PSQI questionnaire assesses sleep quality in the past month [39]. This index has been widely used among general and clinical populations across countries. The items, each rated on a 0–3 scale, are grouped into seven components. The total scores range from 0 to 21, with a higher score indicating poorer sleep quality [39]. The internal consistency of the traditional Chinese PSQI is satisfactory (Cronbach’s alpha = 0.75).

#### ENRICHD social support instrument (ESSI)

The traditional Chinese ESSI was used to measure social support. The 6-item scale has a global score from 6 to 30. A higher score denotes a higher level of social support [40]. The traditional Chinese ESSI had a Cronbach’s alpha of 0.79 [40].

#### Patient health questionnaire (PHQ-15)

The traditional Chinese PHQ-15, which has been validated in the Chinese population, was applied to measure psychosomatic symptoms [41]. It comprises 15 somatic symptoms, and each was rated from 0 (not bothered at all) to 2 (bothered a lot). Cronbach’s alpha of the traditional Chinese PHQ-15 was reported to be 0.79 [41].

#### Perceived stress scale (PSS)

We administered the traditional Chinese 10-item PSS to evaluate stress levels. The 10-item version, which has two components of stress, showed better validity and reliability than the 14-item and 4-item PSS [42]. Internal consistency, assessed by Cronbach’s alpha of the traditional Chinese 10-item PSS, was 0.85 [42].

#### Hospital anxiety and depression scale (HADS)

The traditional Chinese HADS was adopted to assess anxiety and depression symptoms. The HADS includes 14 items with non-overlapping 7 items assessing anxiety, and 7 items assessing depression. The anxiety and depression subscales were scored independently, and the total scores of the two subscales were both between 0 and 21. A higher total score denotes more severe anxiety or depression [43]. Values of Cronbach’s alpha for the anxiety and depression subscales were 0.80 and 0.63, respectively [43].

### Statistical analysis

Multiple linear regression models with increasing level of covariate adjustment were conducted. Specifically, we first conducted the crude model (Model 1) without any adjustment and Model 2 with adjustment of potential confounders, which included age, gender, education, occupation, income, house type, and marital status. Then, we also conducted Model 3 with additional adjustment for exercise, smoking, alcohol, and BMI (excluded when BMI was the outcome), Model 4 that further adjusted for diagnosis of hypertension (excluded when BMI was the outcome) and the psychometric constructs, and Model 5 that also adjusted for sleep parameters. The analysis was also stratified by gender. To assess the robustness of the results, we also conducted sensitivity analysis by repeating the analysis on people without hypertension, on people without taking antihypertensive medications, and on people living in multi-story buildings. The presence of multicollinearity was assessed using the variance inflation factor (VIF). A VIF > 5 was considered an indication of the presence of multicollinearity [44]. An interaction of noise by sociodemographic and lifestyle characteristics, and noise sensitivity, respectively, was added separately to Model 2 to test the moderating role. An overall *p*-value was reported if the interaction of noise by categorical variables (marital status, smoking, and alcohol) was tested. We assessed mediators in parallel using the product-of-coefficients method with package “mediation” based on Model 2 [45, 46]. Indirect effects were tested with no strict limitation on the associations of the mediators (sleep duration and sleep quality) with the independent variables and with the dependent variables [47]. The effect of every 10 dBA increase of nocturnal noise was reported. Model adequacy was tested using the normal probability plots and scatter plots of residuals against the predicted values. Estimates were accompanied by 95% confidence intervals (CIs) where appropriate, and 5% nominal level of significance was used in all significance tests. Regression analyses were conducted using RStudio-1.2.1335.

## Results

### Characteristics

A total of 500 adults participated in this study and completed the assessments. Table 1 shows summary data including sociodemographic, diagnosis of hypertension, lifestyle, nocturnal noise level, BMI, blood pressure, and scale score details. The average patient age was 39.0 years (range: 18–80 years). Overall, 473 participants lived in multi-story buildings, and 27 lived in townhouses.

**Table 1:** Summary of social-demographic characteristics, nocturnal noise, BMI, blood pressure, and scale scores of the 500 participants

Characteristics	Mean ± SD/n	%
Mean age ± SD (years)	39 ± 12	/
Gender		
Male	168	34%
Female	332	66%
Marital status		
Single	171	34.4%
Married/Cohabiting	307	61.2%
Separated/Divorced/Widowed	22	4.4%
Educational level (1 missing)		
Grade 6 or below	24	5%
Grade 7–12	251	50%
Bachelor or above	224	45%
Occupation		
Working	370	74%
Not working	73	14%
Retired	15	3%
Students	42	8%
Family income (Hong Kong Dollars, 10 missings)		
< 5000	10	2%
5000–9999	7	1.4%
10,000–14,999	17	3.4%
15,000–19,999	29	5.8%
20,000–24,999	46	9.2%
25,000–29,999	53	10.6%
30,000–34,999	68	13.6%
35,000–39,999	37	7.4%
40,000–44,999	55	11.0%
45,000–49,999	37	7.4%
> 50,000	131	26.2%
House type		
Multi-story buildings	473	95%
Townhouse	27	5%
Diagnosis of hypertension		
No	478	96%
Yes	22	4%
Aerobic exercise per week		
< 1 h	170	34%
1 h	116	23%
2 h	88	18%
3 h	61	12%
4 h	28	6%
5 h or more	37	7%
Smoking		
Never	404	81%
Quit	38	7%
Yes	58	12%
Alcohol		
Never	270	54%
Quit	36	7%
Yes	194	39%
Nocturnal noise level (dBA)	51.32 ± 5.61	/
BMI (kg/m^2^)	22.31 ± 3.66	/
SBP (mmHg)	116.16 ± 12.17	/
DBP (mmHg)	75.44 ± 7.97	/
TST (min)	345.1 ± 57.38	/
PSQI	5.14 ± 2.73	/
WNSS	60.44 ± 12.09	/
ESSI	21.39 ± 4.76	/
PHQ	3.93 ± 3.88	/
PSS	15.60 ± 5.31	/
Anxiety	4.43 ± 3.47	/
Depression	5.15 ± 3.44	/

*SD* Standard deviation, *BMI* Body mass index, *SBP* systolic blood pressure, *DBP* diastolic blood pressure, *TST* total sleep time, *PSQI* Pittsburgh Sleep Quality Index, *WNSS* Weinstein Noise Sensitivity Scale, *ESSI*: ENRICHD Social Support Instrument, *PHQ* Physical Health Questionnaire, *PSS* Perceived Stress Scale

### Associations of nocturnal noise with BMI and blood pressure

Unadjusted effects of per 10 dBA increase of nocturnal noise exposure level on BMI, SBP, DBP were 0.61 kg/m^2^ (95% CI: 0.07 to 1.13, *p* = 0.025), 3.03 mmHg (95% CI: 1.14 to 4.92, *p* = 0.002), and 0.68 mmHg (95% CI: − 0.65 to 2.01, *p* = 0.317), respectively. Table 2 shows that after adjusting for sociodemographic characteristics, nocturnal noise was significantly associated with BMI (b = 0.54, 95% CI: 0.01 to 1.06, *p* = 0.045) and SBP (b = 2.90, 95% CI: 1.12 to 4.68, *p* = 0.001). No significant association was detected between nocturnal noise and DBP (b = 0.79, 95% CI: − 0.56 to 2.13, *p* = 0.253). Nocturnal noise remained to be associated with SBP (b = 2.41, 95% CI: 0.62 to 4.20, *p* = 0.008), but the associations with BMI (b = 0.50, 95% CI: − 0.03 to 1.03, *p* = 0.064) and DBP (b = 0.52, 95% CI: − 0.88 to 1.92, *p* = 0.465) were not statistically significant after additionally adjusting for lifestyle, BMI (excluded when BMI was the outcome), noise sensitivity, somatic symptoms, stress, anxiety, depression, diagnosis of hypertension (excluded when BMI was the outcome), objective total sleep duration, and subjective sleep quality. VIF values for the SBP model ranged from 1.03 to 1.56.

**Table 2:** Estimated effects of nocturnal noise level from multiple linear regression of BMI, SBP, and DBP in Chinese Adults

	BMI		SBP		DBP	
	Estimate (95% CI)	*p*-value	Estimate (95% CI)	*p*-value	Estimate (95% CI)	*p*-value
**Model 1**	0.61 (0.07, 1.13)	0.025	3.03 (1.14, 4.92)	0.002	0.68 (−0.65, 2.01)	0.317
**Model 2**	0.54 (0.01, 1.06)	0.045	2.90 (1.12, 4.68)	0.001	0.79 (−0.56, 2.13)	0.253
**Model 3**	0.51 (−0.02, 1.03)	0.059	2.72 (0.90, 4.53)	0.003	0.69 (− 0.69, 2.07)	0.328
**Model 4**	0.51 (−0.02, 1.04)	0.060	2.64 (0.86, 4.42)	0.004	0.56 (−0.82, 1.95)	0.424
**Model 5**	0.50 (−0.03, 1.03)	0.064	2.41 (0.62, 4.20)	0.008	0.52 (−0.88, 1.92)	0.465

Model 1: crude estimate without adjustment; Model 2: adjusted for age, gender, education, occupation, income, house type, marital status; Model 3: additionally adjusted for exercise, smoking, alcohol, and BMI (excluded when BMI was the outcome); Model 4: additionally adjusted for diagnosis of hypertension (excluded when BMI was the outcome) and the psychometric constructs; Model 5: additionally adjusted for sleep duration and sleep quality

*BMI* body mass index, *SBP* systolic blood pressure *DBP* diastolic blood pressure

### Stratified and sensitivity analysis of nocturnal noise on BMI and blood pressure

Table 3 shows the associations of nocturnal noise with BMI, and with blood pressure in different groups. Nocturnal noise was not associated with DBP in all subgroups with *p*-values greater than 0.171. Every 10 dBA increase in nocturnal noise was associated with 0.72 kg/m^2^ increase in BMI (95% CI: 0.07 to 1.38, *p* = 0.031) and 3.91 mmHg increase in SBP (95% CI: 2.51 to 5.31, *p* < 0.001) in females and 3.13 mmHg increase in SBP (95% CI: 1.35 to 4.92, *p* < 0.001) in males. Moreover, for people without diagnosis of hypertension, without taking medication for hypertension, living in the multi-story buildings, every 10 dBA increase in nocturnal noise was significantly associated with 3.54 mmHg (95% CI: 2.40 to 4.67, *p* < 0.001), 3.58 mmHg (95% CI: 0.75 to 4.39, *p* < 0.001), and 3.01 mmHg (95% CI: 1.20 to 4.82, *p* = 0.001) increase in SBP, respectively.

**Table 3:** Stratified and sensitivity analysis on the associations between nocturnal noise level and BMI, SBP and DBP

	BMI		SBP		DBP	
	Estimate(95% CI)	*p*-value	Estimate(95% CI)	*p*-value	Estimate(95% CI)	*p*-value
**Male** ^**a**^	0.27(−0.63, 1.18)	0.552	3.13 (1.35, 4.92)	< 0.001	0.43(−1.98, 2.85)	0.724
**Female** ^**a**^	0.72 (0.07, 1.38)	0.031	3.91 (2.51, 5.31)	< 0.001	1.15(−0.50, 2.81)	0.171
**Without diagnosis of hypertension** ^**b**^	/	/	3.54 (2.40, 4.67)	< 0.001	0.34(−1.03, 1.71)	0.625
**Without taking medication** ^**b**^	/	/	3.58 (0.75, 4.39)	< 0.001	0.38(−0.98, 1.74)	0.582
**Multi-storey buildings**	0.49(−0.03, 1.02)	0.066	3.01 (1.20, 4.82)	0.001	0.75(−0.64, 2.13)	0.290

*BMI* body mass index, *SBP* systolic blood pressure *DBP* diastolic blood pressure

^a^: adjusted for age, education, occupation, income, house type, marital status;

^b^: adjusted for age, gender, education, occupation, income, house type, marital status

### Moderating effects of sociodemographic, lifestyle, and noise sensitivity on the associations between noise and BMI and blood pressure

We assessed the moderating effects of sociodemographic, lifestyle, and noise sensitivity on BMI, SBP, or DBP. Only the moderation of noise by age (b = 0.01, 95% CI: 0.00, 0.02, *p* = 0.009) was observed (Table 4). The *p*-value for the interaction between noise and noise sensitivity on DBP was 0.056.

**Table 4:** Moderating effects of sociodemographic and lifestyle variables and noise sensitivity on nocturnal noise level

	BMI	SBP	DBP
	*p*-value	*p*-value	*p*-value
**Age** ^**a**^	0.640	0.102	0.009
**Gender** ^**a**^	0.381	0.553	0.431
**Marital status** ^**a**^	0.421	0.927	0.148
**Exercise** ^**b**^	0.606	0.291	0.310
**Smoking** ^**c**^	0.177	0.132	0.118
**Alcohol** ^**d**^	0.367	0.907	0.501
**Noise sensitivity** ^**e**^	0.246	0.805	0.056

*BMI* body mass index, *SBP* systolic blood pressure *DBP* diastolic blood pressure

^a^: adjusted for age, gender, education, occupation, income, house type, marital status, noise

^b^: adjusted for age, gender, education, occupation, income, house type, marital status, noise, exercise

^c^: adjusted for age, gender, education, occupation, income, house type, marital status, noise, smoking

^d^: adjusted for age, gender, education, occupation, income, house type, marital status, noise, alcohol

^e^: adjusted for age, gender, education, occupation, income, house type, marital status, noise, noise sensitivity

### Mediating effects of sleep parameters on the associations between noise with BMI and blood pressure

The indirect effects of sleep duration on the associations between noise and BMI, SBP, and DBP were 0.02 (95% CI: − 0.06, 0.01, *p* = 0.602), 0.15 (95% CI: − 0.08, 0.50, *p* = 0.240), and 0.04 (95% CI: − 0.15, 0.30, *p* = 0.680), respectively.

The indirect effects of sleep quality on the associations between noise and BMI, SBP and DBP were − 0.02 (95% CI: − 0.08, 0.00, *p* = 0.382), − 0.02 (95% CI: − 0.19, 0.10, *p* = 0.830), and − 0.04 (95% CI: − 0.19, 0.00, *p* = 0.470), respectively.

## Discussion

To the best of our knowledge, this is the first study to investigate the relationships of nocturnal noise level with BMI and with blood pressure in community-dwelling Chinese adults living in multi-story buildings. We demonstrated that an increase in nocturnal noise exposure measured over one week was associated with higher BMI and blood pressure in the daytime.

Previous studies showed that an increase in exposure to modeled noise levels was significantly associated with higher BMI and WC and a higher risk of obesity [33, 48]. However, Pyko et al. [49] did not find an association between road traffic noise and BMI, but the association with WC as well as central obesity was significant. The effect of noise on obesity was hypothesized through stress response with the activation of the HPA axis, which could lead to increased levels of stress hormones such as cortisol [19, 50]. Alterations in cortisol levels are related to metabolic changes, including weight gain [51]. The association between noise and BMI was only found in females in this study. In an earlier study, an association between noise and obesity was found in women who were highly sensitive to noise [48]. Specifically, every 10 dB increase in road traffic noise was associated with 0.02 and 0.01 units increase in BMI and WC, respectively, and an odds ratio of 1.24 for WHR ≥ 0.85 in highly noise-sensitive women [48]. We posited that females might be more annoyed by noise than males [52]. Therefore, future studies incorporating noise annoyance or other reactions and emotions to noise are desirable to understand the underlying mechanism.

Although the relationship between noise exposure and blood pressure has been extensively investigated, the results, to date, are still inconsistent. Concerning the association between transportation noise and the prevalence of hypertension, the relative risks (RRs) per 10 dBA of road traffic noise, aircraft noise, and rail traffic noise were all approximately 1.05 [1]. However, some studies failed to find the significant associations between noise and blood pressure in specific population groups such as general women [53] and pregnant women [54]. The inconsistencies may result from the sample sizes, self-reported high blood pressure diagnosis, accurate exposure levels of noise, and other potential confounding factors. This study demonstrated the significant association between noise and SBP using measured noise levels and measured blood pressure after adjusting for various confounding factors. This result was consistent with some earlier studies [55, 56]. Moreover, this study revealed that the association between noise and SBP was stronger in females than in males, which was in line with a previous cohort study of 701,174 residents [57]. It also demonstrated no statistically significant association between noise and DBP, which was consistent with one previous study [34]. The associations remained similar when excluding people with hypertension or who were taking medications for hypertension. The difference between the associations of noise with SBP and with DBP might be due to that SBP was more sensitive to noise level [58]. Furthermore, there could be a threshold noise level for DBP [59]. When individuals were exposed to a level lower than 80 dB, DBP did not show a significant increase [60]. The mean nocturnal noise level in this study was around 51 dBA which might be relatively low to cause a significant increase in DBP. On the contrary, a significant association was also reported [29]. Standardized acoustic stressors and time-dependent analysis might be vital for interpreting the association [58]. Therefore, the inconsistent results call for more precise and professional research. Nevertheless, the association between SBP and the risk of CVD calls for the control of potential risk factors [61].

Although the impact of noise on human beings has been found in some specific groups such as females, people who drink alcohol, and highly noise-sensitive people [15, 16, 48], this study did not find any moderating effects of sociodemographic and lifestyle variables, and noise sensitivity, except for the moderating effect of aging on the association between noise and DBP. For the insignificant associations, it might be due to the difference between acute noise and chronic noise and the threshold of the noise level. Nevertheless, the impact of noise on DBP increases as people aging. The previous study indicated that the impact of noise could and should be differentiated between various groups such as children and the elderly [62]. Age might moderate the vulnerability to noise on specific health outcomes given specific groups. It was reported that isolated diastolic hypertension was more common in middle-aged groups in the Chinese population [63]. The mean age of the sample in this study was 39 years, and the middle-aged participants occupied around 50% of the sample. We proposed that age might also moderate the effect of noise on SBP, and a sample incorporating more elderly would be of interest due to the characteristics of systolic blood pressure. Therefore, attention needs to be paid to the effect of age and clarify the possible associations and mechanisms.

It has been hypothesized that noise may affect cardiovascular and metabolic functions through sleep disturbance, where sleep plays a significant role in modulating hormonal release [49]. However, neither objective sleep parameter nor subjective sleep parameter was identified as the mediator in the association between noise and blood pressure or BMI. The effect of noise on hypertension has been hypothesized to be through the activation of the HPA axis [50]. Sleep deprivation is associated with the activation of the HPA axis [64]. Sleep restriction was shown to be associated with increased sympathetic activity and venous endothelial dysfunction [65]. Hence, people who have sleep disturbance have higher risks of hypertension. In terms of BMI, previous studies have indicated that lack of sleep increases appetite and reduces energy expenditure by affecting serum levels of leptin and ghrelin, thus increasing the risk of being overweight and obese [66]. The dysregulation of “hunger hormones” due to the anorexigenic hormone leptin levels resulting from sleep disturbances may induce increased food intake [67]. Sleep deprivation may lead to reduced energy expenditure through altered thermoregulation and increased fatigue [68]. Hence, the risk of being overweight and obese increases. Nonetheless, important factors such as diet were not investigated in this study. Therefore, future studies cover more sleep parameters and confounding factors are needed to verify how sleep affects the relationship between noise and health problems.

This study used a noise dosimeter to record nocturnal noise levels for a week, which was much more precise than the estimated noise levels used previously. Furthermore, both subjective sleep and objective sleep parameters were included. In addition, noise sensitivity, considered to affect vulnerability, was studied.

However, several limitations are worth noting. First, body weight and height measurements were self-reported by the participants, which might have led to imprecision. WC and laboratory indicators of obesity could also be good indices. Second, we did not include eating habits in this study, yet we were evaluating effects on BMI. Third, blood pressure was not measured at the same time in the participants, which might have affected the results. However, these might not be very feasible in a community setting. The time of blood pressure measurements needs to be recorded in the future, and any differences in neighborhood noise from other noise sources need to be noted. Fourth, future studies need to include the family history of hypertension, considering its predictive role in blood pressure and obesity [69]. Fifth, this study only measured A-weighted sound levels. Future studies including more characteristics such as frequency and variations are desirable [70, 71]. Lastly, the sample power might not be large enough to examine certain effects, such as the interaction, and studies with a larger sample size are of interest.

## Conclusions

In this study, higher nocturnal noise was associated with higher BMI and SBP, while no statistically significant relationship between noise and DBP was observed. However, the association between noise and BMI was only found in females. The impact of noise pollution on health calls for further study, and controlling nocturnal noise level by using soundproofing materials, noise cancellation devices, or earplugs may contribute to a reduction of related health risks.

## Data Availability

The datasets generated and/or analyzed during the current study are not publicly available due to confidentiality. The data access needs approval from the institutional and ethics committee.

## Abbreviations

BMI: Body mass index
CI: Confidence interval
CVD: Cardiovascular disease
DBP: Diastolic blood pressure
HADS: Hospital Anxiety and Depression Scale
HPA: Hypothalamic-pituitary-adrenal
PHQ-15: Patient Health Questionnaire
PSQI: Pittsburgh Sleep Quality Index
PSS: Perceived Stress Scale
RR: Relative risk
SBP: Systolic blood pressure
VIF: Variance inflation factor
WC: Waist circumference
WNSS: Weinstein Noise Sensitivity Scale
